# Distinct Localization of SNAP47 Protein in GABAergic and Glutamatergic Neurons in the Mouse and the Rat Hippocampus

**DOI:** 10.3389/fnana.2017.00056

**Published:** 2017-07-13

**Authors:** Agnieszka Münster-Wandowski, Heike Heilmann, Felix Bolduan, Thorsten Trimbuch, Yuchio Yanagawa, Imre Vida

**Affiliations:** ^1^Institute of Integrative Neuroanatomy, Charité—Universitätsmedizin Berlin Berlin, Germany; ^2^Institute of Neurophysiology, Charité—Universitätsmedizin Berlin Berlin, Germany; ^3^Departments of Genetic and Behavioral Neuroscience, Gunma University, Graduate School of Medicine Maebashi City, Japan; ^4^Neurocure Cluster of Excellence, Charité—Universitätsmedizin Berlin Berlin, Germany

**Keywords:** SNAP47, hippocampus, GABAergic cells, synaptic localization, mossy fiber projection, immunoelectron microscopy, *in situ* hybridization

## Abstract

Synaptosomal-associated protein of 47 kDa (SNAP47) isoform is an atypical member of the SNAP family, which does not contribute directly to exocytosis and synaptic vesicle (SV) recycling. Initial characterization of SNAP47 revealed a widespread expression in nervous tissue, but little is known about its cellular and subcellular localization in hippocampal neurons. Therefore, in the present study we applied multiple-immunofluorescence labeling, immuno-electron microscopy and *in situ* hybridization (ISH) and analyzed the localization of SNAP47 in pre- and postsynaptic compartments of glutamatergic and GABAergic neurons in the mouse and rat hippocampus. While the immunofluorescence signal for SNAP47 showed a widespread distribution in both mouse and rat, the labeling pattern was complementary in the two species: in the mouse the immunolabeling was higher over the CA3 *stratum radiatum*, *oriens* and cell body layer. In contrast, in the rat the labeling was stronger over the CA1 neuropil and in the CA3 *stratum lucidum*. Furthermore, in the mouse high somatic labeling for SNAP47 was observed in GABAergic interneurons (INs). On the contrary, in the rat, while most INs were positive, they blended in with the high neuropil labeling. ISH confirmed the high expression of SNAP47 RNA in INs in the mouse. Co-staining for SNAP47 and pre- and postsynaptic markers in the rat revealed a strong co-localization postsynaptically with PSD95 in dendritic spines of pyramidal cells and, to a lesser extent, presynaptically, with ZnT3 and vesicular glutamate transporter 1 (VGLUT1) in glutamatergic terminals such as mossy fiber (MF) boutons. Ultrastructural analysis confirmed the pre- and postsynaptic localization at glutamatergic synapses. Furthermore, in the mouse hippocampus SNAP47 was found to be localized at low levels to dendritic shafts and axon terminals of putative INs forming symmetric synapses, indicating that this protein could be trafficked to both post- and presynaptic sites in both major cell types. These results reveal divergent localization of SNAP47 protein in mouse and rat hippocampus indicating species- and cell type-specific differences. SNAP47 is likely to be involved in unique fusion machinery which is distinct from the one involved in presynaptic neurotransmitter release. Nonetheless, our data suggest that SNAP47 may be involved not only postsynaptic, but also in presynaptic function.

## Introduction

Soluble N-ethylmaleimide-sensitive factor attachment protein (SNAP) receptors, also called SNAREs, play a crucial role in synaptic transmission as central components of the fusion machinery (Südhof, 2004). SNAREs comprise two main groups of conserved membrane-associated proteins: the v-SNAREs (“v” for vesicular) VAMP/synaptobrevins and the t-SNAREs (“t” for target) syntaxins and SNAPs (Hohenstein and Roche, 2001). The SNAP family contains four members: SNAP25 (Jahn et al., 2003; Jahn and Scheller, 2006), SNAP23 (Ravichandran et al., 1996; Wang et al., 1997), SNAP29 (Steegmaier et al., 1998); and Synaptosomal-associated protein of 47 (SNAP47; Holt et al., 2006). SNAP47 is the most recently identified neuronal SNAP which shows a widespread distribution on intracellular membranes, including synaptic vesicles (SVs), but also other intracellular membrane pools in the brain (Holt et al., 2006; Takamori et al., 2006).

The different SNAP isoform proteins display distinct patterns of distribution in different neuronal and non-neuronal populations in the central nervous system (CNS). They are strongly expressed in excitatory (glutamatergic) synapses, pre- as well as postsynaptically. The best characterized SNAP25 and SNAP23 proteins are preferentially and abundantly expressed presynaptically in vesicular glutamate transporter 1 (VGLUT1)-positive excitatory terminals in the hippocampus (Verderio et al., 2004; Garbelli et al., 2008) and in vesicular glutamate transporter 2 (VGLUT2)-positive terminals in the neocortex (Bragina et al., 2007), respectively. It has been also reported that hippocampal and cortical inhibitory interneurons (INs) may selectively express SNAP23 (Verderio et al., 2004; Bragina et al., 2007), however, there is very little data regarding the subcellular localization of specific SNAP isoforms in these neurons. These two SNAP isoforms were also found postsynaptically in spines at substantially lower levels. Although their postsynaptic function has not yet been fully identified, SNAP25 is involved in the regulation of spine formation (Tomasoni et al., 2013) whereas SNAP23 contributes to glutamate receptor trafficking (Suh et al., 2010).

In contrast to SNAP25 and SNAP23, SNAP47 do not contain palmitoylated cysteine clusters, important for the localization to surface membranes. This SNAP, thus, may be involved in intracellular vesicle trafficking and fusion events (Kuster et al., 2015), consistent with a wider subcellular distribution (Holt et al., 2006). Recently, Jurado et al. (2013) documented postsynaptic dendritic localization of SNAP47 and suggested an involvement in postsynaptic fusion events in glutamatergic neurons in the hippocampus. However, SNAP47 is not exclusively localized in postsynaptic structures (Holt et al., 2006). In a recently published study, Shimojo et al. (2015) found endogenous SNAP47 distribution to axons in cortical neuronal cultures, as well as, in native tissue. Thus, existing data suggest both pre- and postsynaptic distribution of this protein, but information regarding the precise subcellular localization of SNAP47 in cortical neurons is still lacking.

Therefore, to address the subcellular distribution of SNAP47 protein and RNA in hippocampal neurons, in this study we applied immunofluorescent, *in situ* hybridization (ISH) and postembedding immunogold labeling combined with confocal- and electron microscopic analysis in mouse and rat hippocampus with a focus on pre- and postsynaptic elements of glutamatergic and GABAergic neurons.

## Materials and Methods

### Animals

A total of three adult male wild-type (WT) C57BL/6J mice (~2.5-month-old, 25–30 g) and three adult male Wistar rats (~3-month-old, 300–350 g) were obtained from the local animal breeding of the Charité, Berlin. In addition, in order to identify cortical GABAergic INs, we used seven adult male transgenic mice and seven adult male transgenic rats expressing improved yellow fluorescent protein (YFP; Venus) under the promoter of the vesicular GABA-transporter (VGAT) of comparable age and weight. The generation of VGAT-Venus transgenic rats and mice has been described previously (Uematsu et al., 2008; Wang et al., 2009). In these transgenic animals, Venus is expressed highly selectively in 95%–98% of cortical GABAergic INs in the neocortex and the hippocampus. VGAT-Venus transgenic mice and rats exhibit otherwise normal growth and reproductive behavior.

Of these animals, all 10 animals (3 WT and 7 transgenic) of both species were used for immunofluorescence labeling; semi-quantitative analysis of SNAP47 labeling intensities were performed in tissue from three transgenic animals of both species. For immunoelectron microscopic analysis two animals and for ISH also two animals of both species were used. All procedures, animal handling and maintenance were performed in accordance with recommendations of the animal welfare committee of the Charité Berlin, Germany; the National Act on the Use of Experimental Animals, Germany; local authorities (LaGeSo Berlin, registration number: O-0098/12) and the European Council Directive 86/609/EEC.

### Tissue Preparation

The animals were anesthetized with a mixture of ketamine 50 mg/kg (Actavis) and xylazine (Rompun) 20 mg/ml (Bayer Health Care, Berlin, Germany) and perfused transcardially with fixative containing 4% paraformaldehyde (PFA, Electron Microscopy Sciences, Hatfield, PA, USA) with 0.2% picric acid (Fluka Chemie, Buchs, Switzerland) in a phosphate buffered solution (0.1 M PB, pH = 7.2). For electron microscopy the fixative contained additionally 0.05%–0.1% glutaraldehyde (GA, Electron Microscopy Sciences). The brains were removed and dissected into blocks containing the hippocampus using a coronal rodent brain matrix (ASI Instruments, Warren, MI, USA) and were processed for light- and electron microscopy. In all experiments, we analyzed 3–5 sections of dorsal hippocampus from each animal.

### Primary Antibodies

For a comprehensive list of the antibodies including their characteristics, dilution and source please see Table 1. Currently only one full length polyclonal antibody against SNAP47 raised in rabbit is available and was used for all double and triple immunolabeling in our study. The antibody against the SNAP47 protein was first used in a single immunofluorescence analysis to determine the distribution profile of this protein in mouse and rat hippocampus. Double and triple-immunostaining for SNAP47 and several specific GABA and glutamatergic cellular and pre- or postsynaptic markers were used to determine the cellular and compartmental distribution of the SNAP47 protein in hippocampus (see Table 1).

**Table 1:** List of primary antibodies for immunocytochemistry.

Antibody	Supplier and cat. no.	Host	Dilution	Immunogen	
**SNARE protein**	
SNAP47	Synaptic System	Rabbit	LM 1:300	Recombinant full length rat SNAP47.
	111 403	(polyclonal, affinity purified)	EM 1:30	
**Pre-synaptic glutamatergic marker proteins**
ZnT3	Synaptic System	Guinea pig	LM 1:300	Recombinant protein of mouse ZnT3.
	197 004	(polyclonal, crude antiserum)		(aa 2–75).
VGLUT1	Synaptic System	Guinea pig	LM 1:2000	Purified recombinant protein of rat
	135 304	(polyclonal, crude antiserum)		VGLUT1 (aa 456–560).
**Post-synaptic glutamatergic marker protein**
PSD95	UC Davis/NIH	Clone K28/43	1:100	Fusion protein amino acids 77–299
	NeuroMab Facility	Mouse		(PDZ domains 1 and 2 of human PSD95).
	75–028	(monoclonal)		
**GFP/YFP**			
GFP	UC Davis/NIH	Clone N86/38	LM 1:2000	Green fluorescent protein against fusion protein amino acids 1–238 (full length) of jellyfish green fluorescent protein.
	NeuroMab Facility	Mouse	EM 1:100
	75–132	(monoclonal)		

### Viral Construct, Cell Culture and Western Blotting Verification of SNAP47 Antibody Specificity

Due to limited commercial availability of SNAP47 antibody, we characterized specificity of polyclonal SNAP47 recombinant full length antibody from Synaptic System applying a lentiviral-based method.

#### Lentiviral Constructs and Virus Production

The coding sequences of mouse SNAP47 (NCBI Reference Sequence NM_144521) and rat SNAP47 (NCBI Reference Sequence: NM_199389) were PCR-amplified from total mouse or rat brain cDNA using custom designed primers: msSNAP47 BsrGI fw (5′-gct gta caa ggg atc cgg aat gag ttc tga tat gcg tgt cc-3′), msSNAP47 AscI rev (5′-aag gcg cgc cct aca tca gct ttc tca tac gc-3′), ratSNAP47 BsrGI fw (5′-gct gta caa ggg atcc gga atg agc agt gat gta cga gtt c-3′), ratSNAP47 AscI rev (5′-aag gcg cgc cct aca aca gct ttc tca tac gac-3′). The resulting PCR products were cloned into a modified lentiviral GFP expressing vector (Lois et al., 2002) which would lead to the translation of a N-terminal GFP tagged mouse or rat SNAP47 protein which is controlled by a human Synapsin-1 promoter to restrict neuronal expression. After sequence verification, lentiviral particles were prepared by the Charité Viral Core Facility as described previously (Lois et al., 2002).

#### Neuronal Culture and Western Blotting

Murine cell cultures were prepared as described (Chang et al., 2014). Primary hippocampal neurons were prepared from mice on embryonic day E18 and plated at 10.000 cm-2 on a continental murine astrocyte feeder layer. N-terminally GFP-tagged mouse and rat SNAP47 was lentivirally expressed within murine neuronal cell cultures to validate the SNAP47 antibody. Neurons were infected at DIV 1 with 5 × 105 − 1 × 106 infectious virus units per 35 mm-diameter well. Protein lysates were obtained at DIV 14. Briefly, cells were lysed using 50 mM Tris/HCl (pH 7.9), 150 mM NaCl, 5 mM EDTA, 1% Triton-X-100, 1% Nonidet P-40, 1% sodium deoxycholate and protease inhibitors (cOmplete protease inhibitor cocktail tablet, Roche Diagnostics GmbH). Proteins were separated by SDS-PAGE and transferred to nitrocellulose membranes. After blocking with 5% milk powder (Carl Roth GmbH) for 1 h at room temperature, membranes were incubated with rabbit anti-SNAP47 (1:1000, Synaptic System 111403), mouse anti-GFP (1:1000, Clontech 632381) and guinea pig anti-Synaptophysin (1:1000, Synaptic System 101004) antibodies overnight at 4°C. After washing and incubation with corresponding horseradish peroxidase-conjugated goat secondary antibodies (all from Jackson ImmunoResearch Laboratories), protein expression levels were visualized with ECL Plus Western Blotting Detection Reagents (GE Healthcare Biosciences).

**Figure 1:**
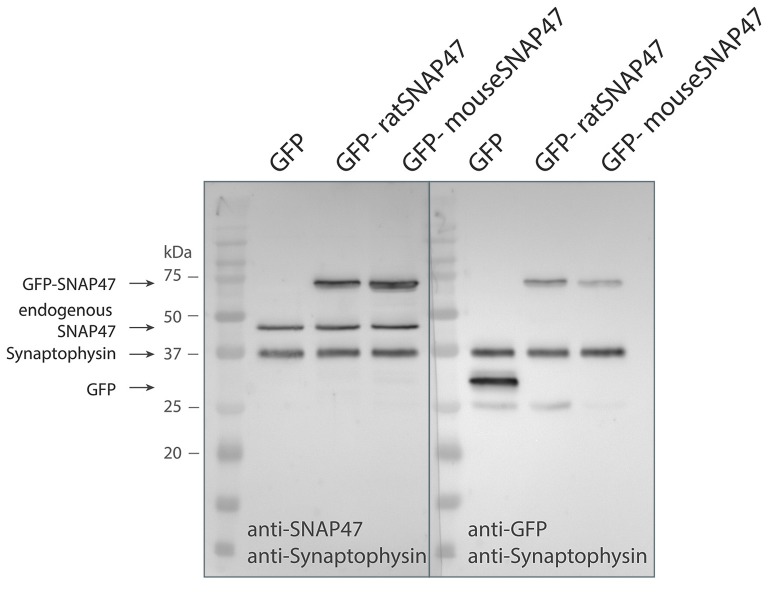
Specificity of the SNAP47 antibody confirmed by the western blot of cultured WT hippocampal neurons infected with lentivirus expressing GFP-tagged mouse or rat SNAP47.Cultured WT hippocampal neurons were infected with either GFP (left lanes), GFP-mouse-SNAP47 (middle) or GFP-rat-SNAP47 (right) expressing lentiviral particles. From these cultures, 30 μg of protein lysates were loaded per lane and subjected to western blotting. The blot on the left was incubated with the rabbit polyclonal anti-SNAP47 antibody, the blot on the right was incubated with an anti-GFP antibody. Synaptophysin (~37 kDa) expression served as internal loading control detected by an anti-synaptophysin antibody. Note that the anti-SNAP47 antibody specifically recognizes the mouse or rat GFP tagged SNAP47 (~74 kDa) as well as the endogenously expressed SNAP47 protein (47 kDa). The anti-GFP antibody in the western blot on the right detects the expression of GFP (~27 kDa) and the GFP-tagged SNAP47 but not the endogenous SNAP47.

Anti-SNAP47 recognizes specifically mouse or rat GFP tagged SNAP47 (app. 72 kDa) and endogenously expressed mouse SNAP47 protein (47 kDa), which is not detected in the GFP control western blot (Figure 1).

### Tissue Processing for Immunofluorescence Labeling

Coronal sections including the hippocampus were cut at 20 μm in a cryostat (CM 3050S; Leica, Wetzlar, Germany). Selected sections from mouse and rat hippocampus were incubated in a mixture of primary antibodies. The double-labeling antibody combinations were the following: GFP + SNAP47, SNAP47 + PSD95, SNAP47 + gephyrin. The triple-labeling antibody combinations were: GFP + SNAP47 + ZnT3, GFP + SNAP47 + VGLUT1, GFP + SNAP47 + VGAT. For detection of the primary antibodies the sections were subsequently incubated in a mixture of the appropriate secondary antibodies: goat anti-mouse (1:300), goat anti-rabbit (1:500), goat anti-guinea pig (1:500) conjugated to Alexa Fluor series fluorochromes. The immunolabeling protocol was performed as previously described (Booker et al., 2013, 2014). Sections were subsequently mounted in Fluorsave mounting medium (Calbiochem, San Diego, CA, USA), coverslipped and examined using a confocal microscope (FV1000, Olympus, Hamburg, Germany, see below).

#### Control Experiments

Since no animals deficient of SNAP47 are available, we carefully validated immunolabeling of SNAP47 focusing on proper negative and positive controls. Negative staining controls for all immunofluorescence procedures were performed by substitution of non-immune serum for the primary or secondary antibodies. As positive control we examined immunofluorescence of SNAP25 protein in hippocampal tissue. To obtain a clear interpretation of the rabbit SNAP47 protein localization, we evaluated first the results of single and double immunostaining for all combinations and compared with those from triple labeling.

### RNA *In Situ* Hybridization (ISH)

In the present study, we aimed to characterize the localization, and expression level of SNAP47 in GABA- and glutamatergic neurons in the hippocampus of mice and rats. To this end, we employed ISH and immunocytochemistry. We first assessed the distribution of SNAP47-positive neurons identified by RNA for SNAP47 and the co-localization with the isoform of SNAP47 protein and VGAT-YFP in mouse and rat hippocampus.

#### Probe Design

For digoxigenin (DIG) ISH, mice and rat brains were cryoprotected (30% sucrose in TRIS-buffered saline), frozen over liquid nitrogen and cut on a cryostat. DIG *in situ* for SNAP47 RNA expression was performed using a 400 bp DNA fragment which comprised the sequence encoding for Y263 to V395 of the SNAP47 protein in the mouse and V255 to L387 in the rat. The Fragment was amplified by PCR from the lentiviral SNAP47 expression vector and cloned into plasmids supplied with the T7 promoter sequence (pSPT18/19), and subjected to an *in vitro* DIG RNA labeling using T7-RNA polymerase in the presence of DIG-UTP.

#### RNA-ISH on Cryosections

Brain sections were washed 5× 5 min in DEPC-PBS to clear cryoprotectant, and dried at 50°C. Next the slices were postfixed in 4% PFA, washed in DEPC-PBS. Then they were acetylated in 0.25% acetic anhydride in 0.1 M triethanolamine-HCl (pH = 8.0) for 10 min and treated with Proteinase K to inactivate rare-cutting restriction enzymes. Sections were pre-hybridized for 15 min at RT in hybridization buffer containing 0.3 M NaCl, 10% Dextran sulfate, 0.02 M Tris (pH = 8.0), 5 mM EDTA (pH = 8.0), 1× Denhardt’s solution, 0.5 mg/ml tRNA, 50% deionized formamide. Following pre-hybridization, they were hybridized by incubating in hybridization buffer containing a DIG-labeled probe for SNAP47 (total conc. 50 ng/μl; 12–20 ng/slice) at 54°C overnight. Post-hybridization washes were performed sequentially 5 × 5 min at 54°C in 5× SSC containing 0.05% Tween at 54°C; 5 × 10 min in 50% formamide/2× SSC at 54°C; 5 × 10 min in 50% formamide/1× SSC at 54°C; 3 × 5 min in 0.1 × SSC at 54°C; 3 × 5 min in 0.1× SSC at RT; 4 × 5 min 1× Tris Puffer containing 0.05% Tween at RT. Next, the slices were incubated for 1 h in blocking buffer containing 4% sheep serum and 1% milk powder, followed by incubating overnight at 4°C with sheep anti-DIG-AP Fab fragment (Roche, Basel, Switzerland) diluted 1:2000 in blocking buffer. The color development of alkaline phosphatase activity was in the presence of two substrates: of 5-bromo-4-chloro-3-indolyl phosphate (BCIP) and nitroblue tetrazolium (NBT), was controlled under RT and stopped by adding in 1× PBS. After washing overnight in 1× PBS, sections were mounted onto slides using Kaisers-Gelantine (Merk, Kat. Nr. 1.09242.0100), coverslipped and examined using an Olympus Microscope and the Metamorph Software. To better compare the distribution of ISH signal to the immunolabeling pattern, sections for ISH were interleaved with section which were processed for immunolabeling for GFP and SNAP47 and examined as described above in “Tissue Processing for Immunofluorescence Labeling” Section.

#### Specificity of Hybridization Probes and the Anti-DIG-AP Antibody

As controls, hybridization with sense probes for mouse and rat and omission of the anti-DIG-AP antibody completely abolished ISH signals and immunoreactivity.

### Confocal Imaging and Quantitative Analysis

In order to get an overview of SNAP47 distribution over the mouse and rat hippocampus, we imaged the 20 μm thick coronal hippocampal sections using an ×4 objective lens on a confocal laser-scanning microscope (Olympus FV1000) and arranged overview images. Higher-resolution images were acquired using an ×60 silicon oil immersion lens, with a zoom factor of either 1 or 4 to resolve individual pre- and postsynaptic puncta. Excitation wavelengths were 488 nm for anti-mouse Alexa Fluor-488 (Invitrogen), 405 nm for anti-guinea pig Alexa Fluor-405 (Jackson Immuno Research, West Grove, PA, USA), 643 nm for anti-rabbit Alexa Fluor-647 (Life Technologies, Darmstadt, Germany) and 515 nm for YFP (Nagai et al., 2002), respectively. The images were acquired through separate channels and temporally non-overlapping excitation of the fluorochromes and analyzed off-line using ImageJ software package (courtesy of W.S. Rasband, U. S. National Institutes of Health, Bethesda, Maryland^1^). The analysis of co-localization was performed in Fiji/ImageJ software based on the isodata algorithm (Ridler and Calvard, 1978) using the auto-threshold plugin.

For the semi-quantitative analysis of SNAP47 proteins in GABAergic neurons of mouse and rat hippocampus, we used three VGAT-Venus transgenic mice and rats from three different litters. Confocal images (nine stacks of each hippocampus) were taken using ×30 objective and analyzed by counting the number of VGAT-Venus positive neurons which were also positive for SNAP47 fluorescence. To compare SNAP47 fluorescence signal intensity in GABAergic INs between mouse and rat hippocampus, we determined the mean labeling intensity over the somata (excluding nucleus) of VGAT-positive neurons and compared these to the mean labeling intensity of the surrounding neuropil in the mouse and the rat hippocampus, respectively. All image processing and analysis were performed using Fiji/ImageJ.

### Electron Microscopy

In order to identify the precise subcellular localization of SNAP47 protein in mouse and rat hippocampal neuron, we combined freeze substitution, low temperature embedding and postembedding immunogold labeling.

#### Freeze Substitution Embedding

One millimeter thick hippocampal slices were cut from previously perfused brains and washed repeatedly (six times 10 min) in 0.1 M PB at pH = 7.2. The slices were cryoprotected with increasing concentrations of saccharose (0.5–2.3 M) dissolved in PB. The tissue was subsequently frozen by plunging it into liquid nitrogen. The samples were then transferred into cold methanol (−90°C) in a freeze-substitution chamber (Leica EM AFS). The methanol was exchanged three times before the specimens were immersed overnight in anhydrous methanol at −90°C, containing 2% (w/v) uranyl acetate (Merck, Darmstadt, Germany). After rinsing several times with methanol, the temperature was gradually raised to −50°C and left overnight. The tissue was then infiltrated with mixtures of Lowicryl HM20 resin (Polysciences, Hirschberg, Germany) and methanol (at proportions of 1:2; 1:1; 2:1, respectively, 1 h each) and finally left in pure resin overnight at −50°C. The samples were transferred to flat embedding molds containing freshly prepared resin at −50°C. UV polymerization was started at −50°C (overnight) and then continued for several days at temperatures gradually increasing from −50°C to −20°C and finally to 20°C. Ultrathin sections (70 nm) were cut on a microtome (Reichert Ultracut S, Leica) and mounted on 200-mesh Formvar-coated nickel grids (Plano, Wetzlar, Germany).

#### Postembedding Immunogold Labeling

All postembedding steps except for the incubation with primary antibodies were performed at room temperature. For single and double immunolabeling with SNAP47 antibody, sections were first incubated two times for 5 min in 0.1 M PBXT (PBS, 0.001% Triton X-100, 0.001% Tween 20, pH = 7.4), followed by 90 min incubation in PBXT supplemented with 2% bovine serum albumin (BSA, Sigma-Aldrich, Darmstadt, Germany) and 5% normal goat serum (NGS; PAN Biotech) at room temperature. The sections were next incubated with primary antibodies diluted in the same buffer overnight at 4°C in a humid chamber. After rinsing several times with PBXT, the binding of primary antibodies was visualized by incubating with goat anti-rabbit or goat anti-guinea pig secondary antibodies conjugated to either 5 or 10 nm gold particles (British BioCell, International, Wetzlar, Germany) in PBXT supplemented with 0.5% acetylated BSA (Aurion, Wageningen, Netherlands), for 90 min in a humid chamber. Grids were rinsed several times in PBXT, PBS, and finally in water. Ultrathin sections were finally stained with 2% aqueous uranyl acetate (Merck, Darmstadt, Germany) for 2 min, and with lead citrate for 30 s. Sections were examined using a Zeiss TEM-912 equipped with a digital camera (Proscan 2K Slow-Scan CCD-Camera, Zeiss, Oberkochen, Germany). For negative controls, primary antibodies were omitted.

#### Quantification of Immunogold Signals

Hippocampal sections embedded in Lowicryl HM20, from two animals, were incubated with the rabbit anti-SNAP47 antibody, and immunogold labeling was detected by secondary goat anti-rabbit antibody coupled to 10 nm gold particles. Quantification was performed using the iTEM software (Olympus GmbH, Münster, Germany). The numbers of gold particles were counted within the defined Region of Interest (ROI) area of two populations of glutamatergic synapses in CA3 region of hippocampus: (1) Mossy fiber boutons (MFBs) and their postsynaptic partners, the CA3 complex spines in *stratum lucidum*; and (2) small boutons and spines forming asymmetric synapses in *stratum radiatum*. Immunopositive (threshold of two gold particles) and negative synaptic compartments were included in the analysis. Density values were finally calculated as the number of gold particles per section area in μm^2^. The labeling density was comparable in two animals. Non-specific labeling (background) was assessed from mitochondria. In MFBs most mitochondria (91.7%) showed no immunogold particles and the rest only a single gold particles. The mean particle density was 2.9 ± 0.8 particle/μm^2^ (204 mitochondria from two animals with an average area of 0.03 ± 0.002 μm^2^). Similarly in the CA3 *stratum radiatum*, the majority of the mitochondria (90%) showed no immunolabeling at all, 10% had only a single particle and the mean density was 2.5 ± 0.8 particle/μm^2^ (100 mitochondria from two animals with an average area of 0.05 ± 0.004 μm^2^).

#### Statistical Analysis

The immunogold particle density over presynaptic boutons, postsynaptic spines and mitochondria (serving as background control), from the *stratum lucidum* and *radiatum* was compared by non-parametric Mann-Whitney test for comparing two group rank differences. Data are reported as mean ± standard error of the mean (SEM). Between-subjects comparisons were considered significant if the *P* value was <0.05.

## Results

### General Distribution Pattern of SNAP47 in the Mouse and the Rat Hippocampus

In order to investigate the cellular localization of SNAP47 in the hippocampus, we first performed immunofluorescence labeling in sections from perfusion-fixed WT mouse and rat brains. The immunolabeling revealed a broad distribution of the protein in hippocampal areas and layers in both species; however, the pattern of SNAP47 distribution was divergent.

**Figure 2:**
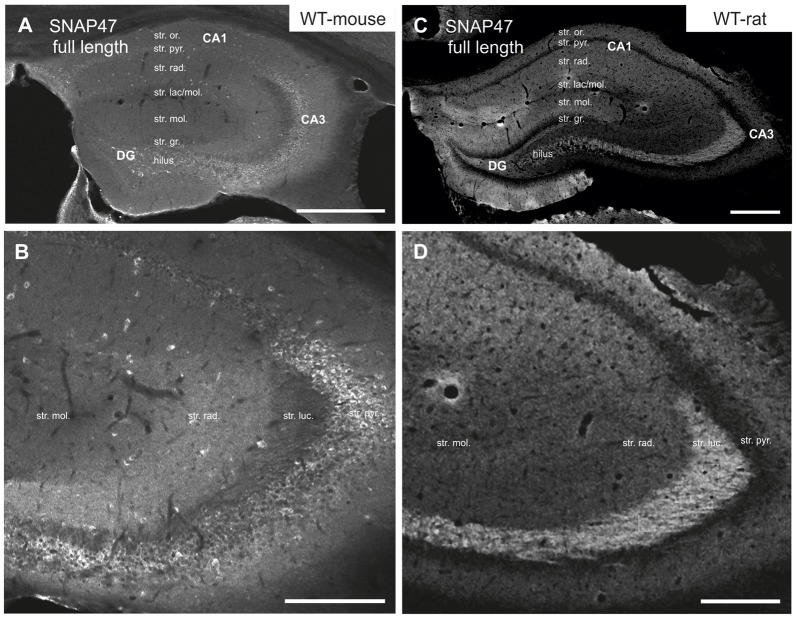
Immunofluorescence labeling pattern of SNAP47 in the hippocampus of WT mice and rats. **(A,B)** Confocal images of SNAP47 immunolabeling in the mouse hippocampus. Note the highly labeled neurons scattered across all areas and layers of the hippocampus. The intensity of SNAP47 is stronger in the cell body layer and *stratum radiatum* of the CA3, contrary to the CA1 area. The lowest immunolabeling of SNAP47 is observed in the CA3 *stratum lucidum*. **(C,D)** Confocal images of SNAP47 immunolabeling in rat hippocampus. Note that in the rat section the scattered neurons are not labeled and the labeling intensity is low in the cell body layers. The high intensity of SNAP47 labeling is observed in the *stratum lucidum* of the CA3 and the *stratum radiatum* of CA1 area. Scale bars represent, **(A–C)**, 500 μm; **(B)**, 200 μm; **(D)**, 275 μm.

In the mouse, stronger SNAP47 immunolabeling was observed over the CA3 *stratum radiatum*, *oriens* and the pyramidal cell layer (Figures 2A,B), whereas the *stratum lucidum* was weakly labeled (Figures 2A,B). In contrast in the CA1 region neuropil labeling in *stratum radiatum* and *oriens* was weak (Figures 2A,B). Finally, in the dentate gyrus (DG) labeling intensity was intermediate with a slightly higher labeling at the border between the molecular and granular cell layers (Figure 2A). Additionally, scattered cell bodies of putative GABAergic INs strongly labeled for SNAP47 could be observed in all areas and layers of the hippocampus (Figures 2A,B).

Intriguingly, SNAP47 showed different and almost complementary labeling pattern in the rat hippocampus. The immunolabeling signal was particularly strong in the CA3 *stratum lucidum* and the hilus of the DG (Figures 2C,D) suggesting that the synapses formed by the glutamatergic mossy fiber (MF) projection contains high level of associated SNAP47 protein. In contrast, the CA3 pyramidal cell layer and *stratum radiatum* were less strongly labeled when compared to the mouse. In the CA1 area, the labeling was much stronger over the neuropil in *stratum radiatum* and *oriens* (Figures 2C,D). Slightly stronger labeling was present in the molecular layer of DG, whereas the granular cell layer was weakly labeled (Figure 2C). Finally, in contrast to mouse, the labeling of scattered INs was absent in the rat hippocampus (Figures 2C,D).

In summary, the labeling for SNAP47 is stronger in the cell body layers and interneuron somata of the mouse hippocampus, suggesting a preferential somatic and postsynaptic localization of this protein in this species. In the rat it showed overall stronger neuropil labeling suggesting preferential localization to the neuronal processes. The complementary distribution of SNAP47 in the mouse and in the rat is semi-quantitatively summarized in Table 2.

**Table 2:** Summary of the SNAP47 protein distribution according to regions and layers in the mouse and the rat hippocampus (SNAP47 immunoreactivity: +++, strong; ++, moderate; +, weak; −, virtually absent).

Hippocampus		Mouse	Rat
Region	Layer		
CA1	oriens	+	++
	pyramidale	++	−
	radiatum	+	++
	lacunosum	+	+
CA3	oriens	++	+
	pyramidale	+++	−
	lucidum	+	+++
	radiatum	++	+
	lacunosum	+	+
DG	molecular layer	+	++
	granular cells	+	−
	hilus	++ (cell bodies)	++
INs		++	+/−

### SNAP47 Co-Localizes with GABAergic INs

The strong labeling of scattered cell bodies in all hippocampal areas and layers in WT mouse suggested high expression of SNAP47 protein in GABAergic INs. In order to confirm this hypothesis, we took advantage of the VGAT-YFP transgenic mouse and rat line selectively expressing YFP-Venus in INs under the VGAT promoter and performed labeling for SNAP47 in these transgenic lines (Uematsu et al., 2008; Wang et al., 2009).

#### High Expression of SNAP47 Protein in VGAT-Positive INs in the Mouse Hippocampus

**Figure 3:**
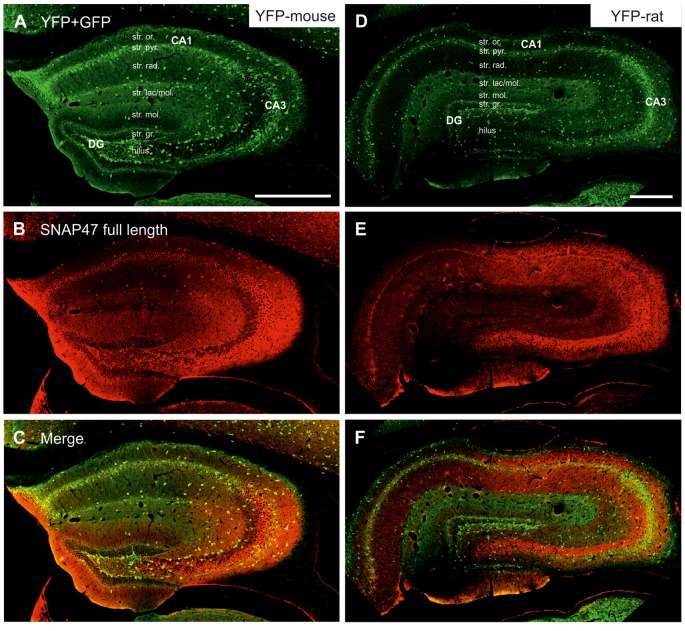
Immunofluorescence labeling pattern of SNAP47 in the hippocampus of VGAT-Venus(yellow fluorescent protein, YFP) transgenic mice and rats. **(A–C)** Confocal images of double immunolocalization for YFP and SNAP47 in the hippocampus of VGAT-Venus(YFP) mouse. Note that the scattered YFP positive interneurons (INs; **A**) are positive for SNAP47 (**B**, superimposed images in **C**). **(D–F)** Confocal images of double immunolocalization for YFP and SNAP47 in the hippocampus of VGAT-Venus(YFP) rat. Note that in contrast to the mouse, the scattered YFP positive INs **(D)** are not strongly labeled for SNAP47 (**E**, superimposed images in **F**) in the rat. Scale bars represent, **(A–F)**, 500 μm.

**Figure 4:**
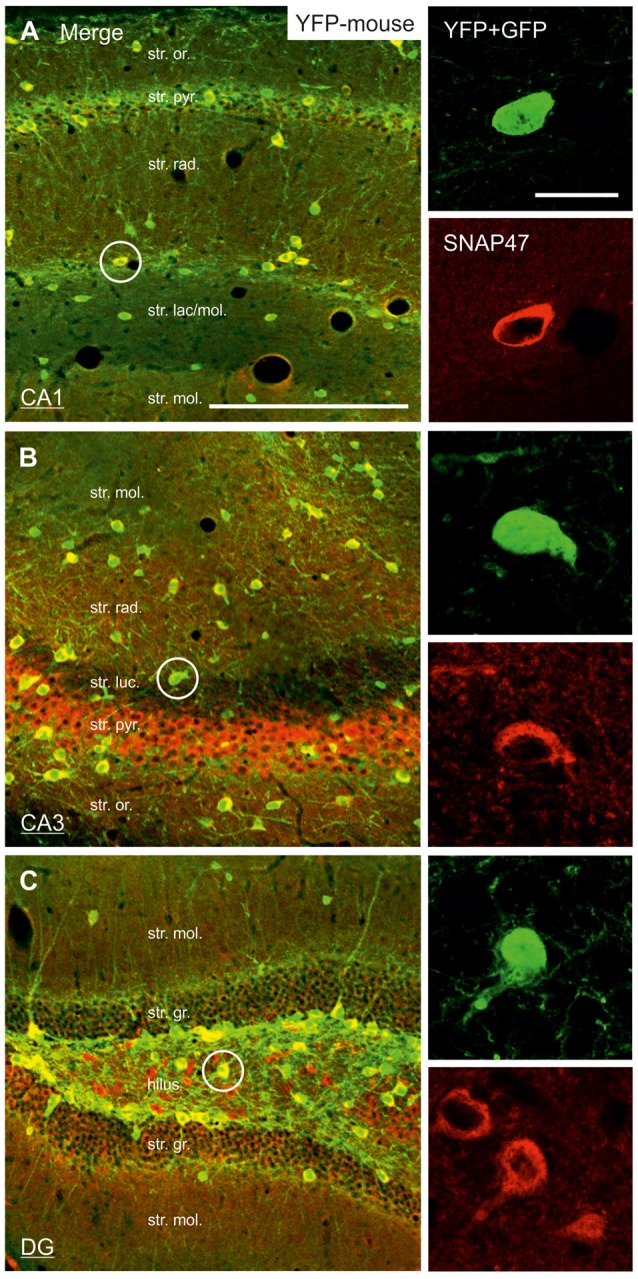
Strong SNAP47 expression in YFP-positive INs in the hippocampus of VGAT-Venus(YFP) transgenic mice. **(A–C)** High-power confocal images of double immunolabeling for YFP and SNAP47 in the CA1 **(A)**, the CA3 areas **(B)** and the dentate gyrus (DG; **C**) of VGAT-Venus(YFP) transgenic mice. Insets on the right show strong cytoplasmic localization of SNAP47 (red pseudocolor, bottom images) in YFP-positive cell bodies (green, top images). Scale bars represent, **(A–C)**, 250 μm; insets, 20 μm.

In good agreement with the labeling observed in the WT mouse, we saw low SNAP47-labeling in the neuropil of the CA1 area (Figures 3B,C, 4A) and *stratum lucidum* of the CA3 (Figures 3B,C, 4B), whereas the labeling was high in the CA3 *stratum radiatum* (Figure 4B), *moleculare* and *pyramidale*, as well as the dentate hilus in sections from transgenic mice (Figures 3B,C, 4C). To facilitate the identification of GABAergic neurons, the YFP signal was amplified by immunostaining using an anti-GFP antibody. In all areas and layers of the hippocampus scattered INs were labeled for YFP (Figure 3C, Supplementary Figure S1) as reported previously (Wang et al., 2009). SNAP47 labeling showed a strong overlap in the somatic cytoplasm of YFP-positive INs (Figures 3A–C, 4A–C and insets). Quantitative data confirmed this observation: in a sample of 537 YFP-positive neurons of the dorsal hippocampi in both hemispheres from three animals essentially all INs were found to be immunopositive for SNAP47. When the mean intensity (arbitrary gray scale units) was assessed in the cytoplasm of YFP-positive cell bodies in the mouse, YFP-positive cell bodies had an almost two-fold higher (239%) mean labeling intensity (2470 ± 76 grayscale value, 50 cells, Figure 6) than the surrounding neuropil in the CA1 *stratum radiatum* (1032 ± 45, Figure 4A with inset, 6A). In the CA3 and the dentate hilus of the mouse, similarly high difference was found between the somatic SNAP47 labeling intensity of YFP-positive cell bodies and the surrounding neuropil (Figures 4B,C with insets, 6B,C).

**Figure 5:**
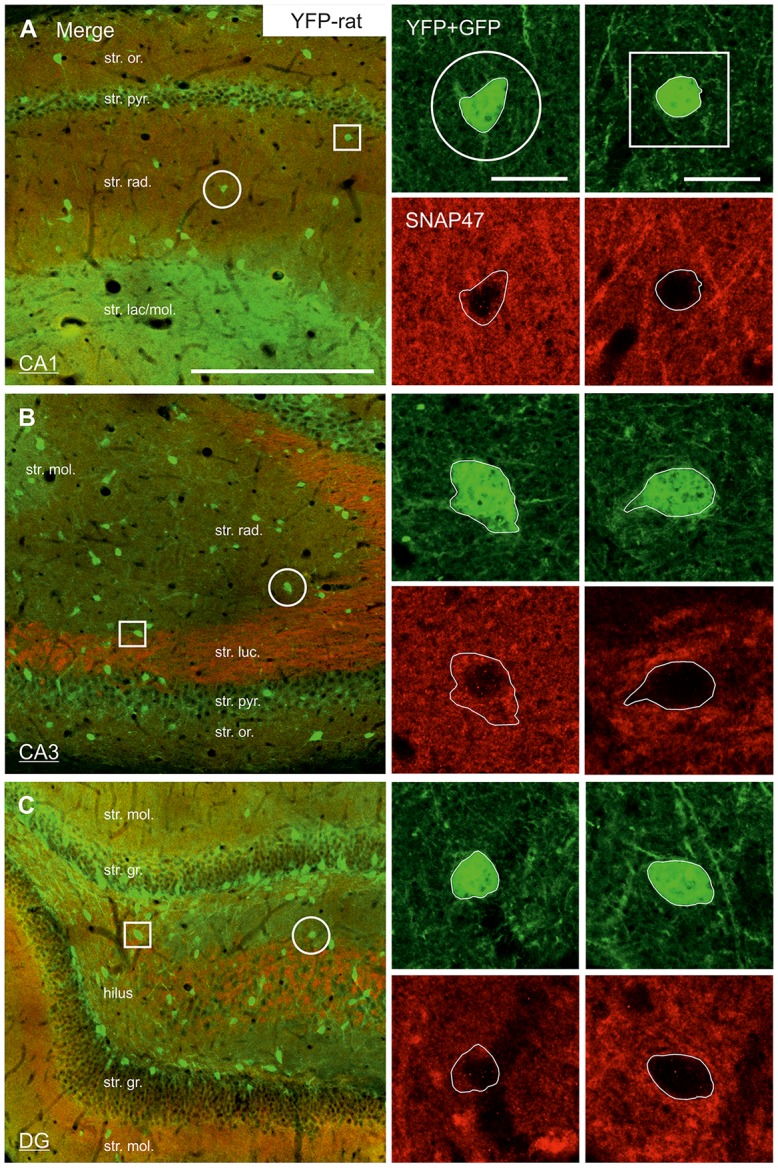
Low SNAP47 expression in YFP-positive INs in the hippocampus of VGAT-Venus(YFP) transgenic rats. **(A–C)** High-power confocal images of double immunolabeling for YFP and SNAP47 in the CA1 **(A)**, the CA3 area **(B)** and the DG **(C)** of VGAT-Venus(YFP) transgenic rats. Insets on the right show SNAP47-positive INs (circles, first column) with weak cytoplasmic labeling (red pseudocolor, bottom images) in the YFP-positive cell bodies (green, top images) and SNAP47-negative INs (squares, second column). Scale bars represent, **(A–C)**, 250 μm; insets, 20 μm.

**Figure 6:**
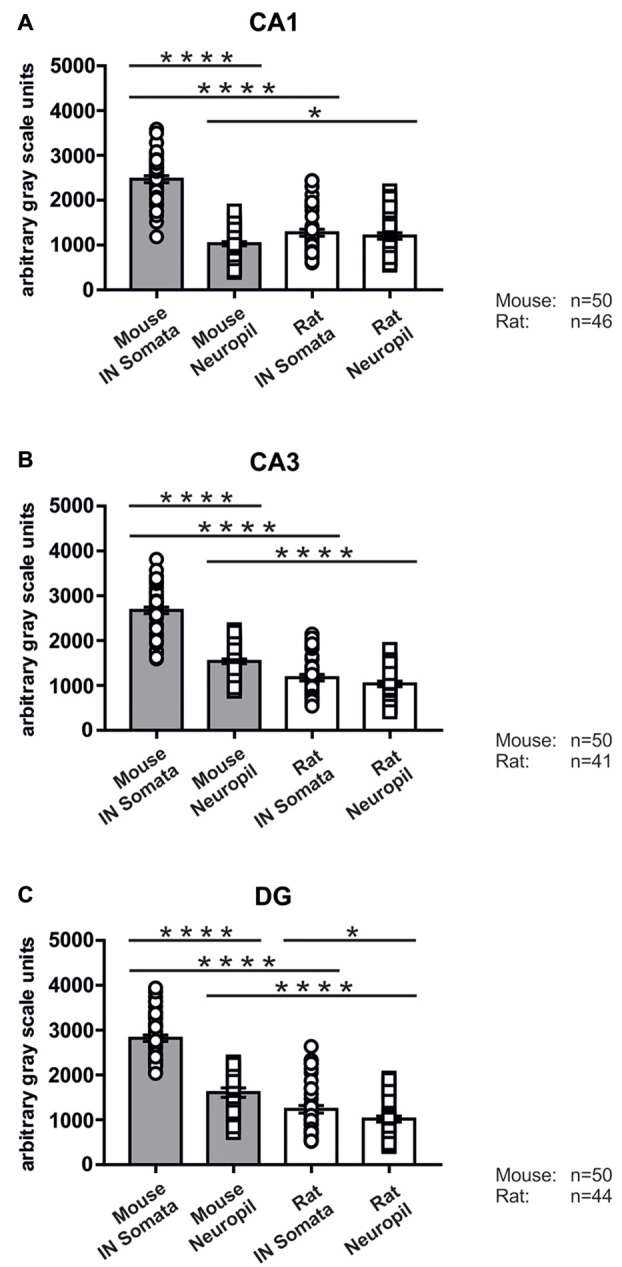
Immunolabeling intensities for SNAP47 in YFP-positive INs and surrounding neuropil in the mouse and the rat hippocampus. Summary bar charts of the immunolabeling intensity for SNAP47 in the somata of YFP-positive INs and surrounding neuropil in the CA1 area **(A)**, the CA3 area **(B)** and the DG **(C)** of the mouse and the rat hippocampus. *P = 0.04, ****P < 0.0001.

#### Low SNAP47 Immunoreactivity in VGAT-Positive INs in the Rat Hippocampus

The lack of the prominent labeling of putative INs in the rat raises the question whether they are devoid of SNAP47. In order to answer this question, we have performed double immunolabeling in a VGAT-YFP transgenic rat line. In good agreement with the labeling in the WT rat and in contrast to that in the mouse, we observed high and homogeneous neuropil labeling in all areas, particularly in the CA1 area of transgenic rats (Figures 3E,F, Supplementary Figure S2). Over the strongly labeled neuropil we could not detect the scattered somata of INs, neither as a higher, positive nor as a lower, negative signal (Figures 3D,E). The expression of SNAP47 in VGAT-positive neurons in the rat was, thus, weaker than in the mouse, but still detectable in most of the INs at a level comparable to the labeling intensity of the surrounding neuropil (Figure 5, insets). Indeed, we found that in the majority of YFP-positive cells (96%, 459 of total 479 cell bodies; from both hippocampi of three animals) showed SNAP47-positive signal in their cytoplasm, only the nucleus was negative (Figure 5, insets panel with circle). When the mean intensity was assessed in the cytoplasm of YFP-positive cell bodies in the rat, it was on average only 6% higher (1275 ± 80 grayscale value, 46 cells, Figure 6A), than the intensity of the surrounding neuropil in the CA1 *stratum radiatum* (1203 ± 72, Figure 5A with insets panel with circle and 6). In other areas, the CA3 and the dentate hilus, similar low difference was found between the SNAP47 labeling intensity in YFP-positive cell bodies and the surrounding neuropil (Figures 5B,C and insets panel with circle, 6B,C), despite the differences in labeling intensities of the neuropil among the areas.

In conclusion, the labeling of the neuropil in the rat obscured the labeling of the cells and made their identification in the SNAP47 labeling difficult. Nevertheless our data indicate that in the mouse essentially all and in the rat hippocampus most INs express SNAP47, however the labeling intensity was strongly different between these two rodent species.

#### SNAP47 RNA-Expressing INs in the Mouse and the Rat Hippocampus

**Figure 7:**
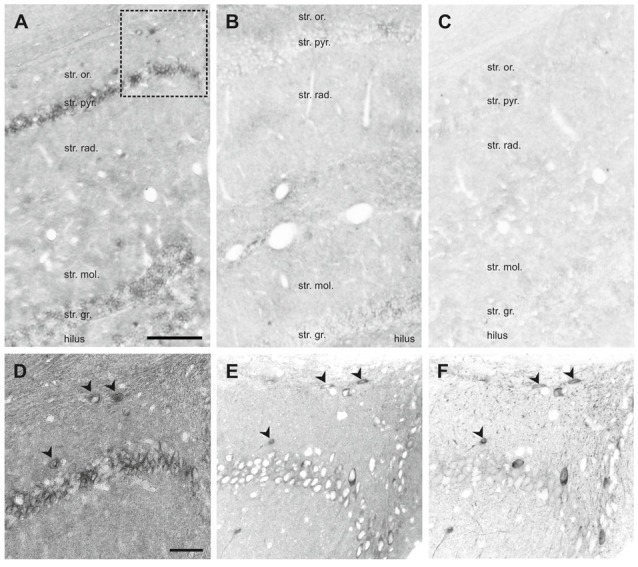
*In situ* hybridization (ISH) of SNAP47 RNA in the mouse and the rat hippocampus. **(A,B)** Overview images ISH with anti-sense probes in mouse **(A)** and rat **(B)** hippocampus. **(C)** ISH image with sense probe in mouse hippocampus. **(D)** Higher magnification images of ISH signal in the *stratum oriens, pyramidale* and *radiatum* of the mouse CA1 area indicated by a box in **(A)**. **(E,F)** Higher magnification images of an adjacent section with double immunofluorescence labeling for SNAP47 **(E)** and GFP **(F)** shown as separate channels and inverted to grayscale. Scale bar represents 100 μm in **(A–F)**.

ISH showed intense cellular expression pattern of SNAP47 RNA in the mouse hippocampus (Figure 7A, Supplementary Figure S3). The pyramidal cell layer in CA1 and CA3 as well as the granular cell layer of DG displayed strong reaction (Figure 7A). We further observed SNAP47 RNA labeling in scattered neurons over all areas and layers of the hippocampus, consistent with the labeling of GABAergic INs (Figure 7A). The highly similar cellular distribution of SNAP47 RNA and the protein in GABAergic cells were confirmed in consecutive slices double-immunolabeled for GFP and SNAP47 (Figures 7D–F).

In the rat, the expression pattern of SNAP47 RNA was generally weaker compared to the mouse. All areas and layers, in particular the cell body layers, showed much lower RNA expression (Figure 7B).

The sense probes used to detect non-specific hybridization exhibited only very low background level of hybridization signal (Figure 7C).

In summary, ISH complemented our immunocytochemical approach and further highlighted: (1) the expression of SNAP47 in principal cells and GABAergic neurons in the mouse hippocampus; and (2) the divergent cellular expression in the mouse and the rat hippocampus.

### SNAP47 Immunoreactivity is Present Both Pre- and Postsynaptically in the Mossy Fiber Projection System

In order to determine whether the high labeling observed in the rat CA3 *stratum lucidum* (Figures 3E,F, 5B) was localized to pre- or postsynaptic elements, we have next performed double labeling for SNAP47 and the Zn-transporter 3 (ZnT3) as a presynaptic marker, known to be highly enriched in MFs and their terminals (Wenzel et al., 1997) or PSD95 as a postsynaptic marker (Kornau et al., 1995; Müller et al., 1996; Rao et al., 1998; Buckby et al., 2004) to identify the complex spines of CA3 pyramidal cells present in this layer.

#### Pre- and Postsynaptic Labeling for SNAP47 in the Mossy Fiber Projection System in the Rat

**Figure 8:**
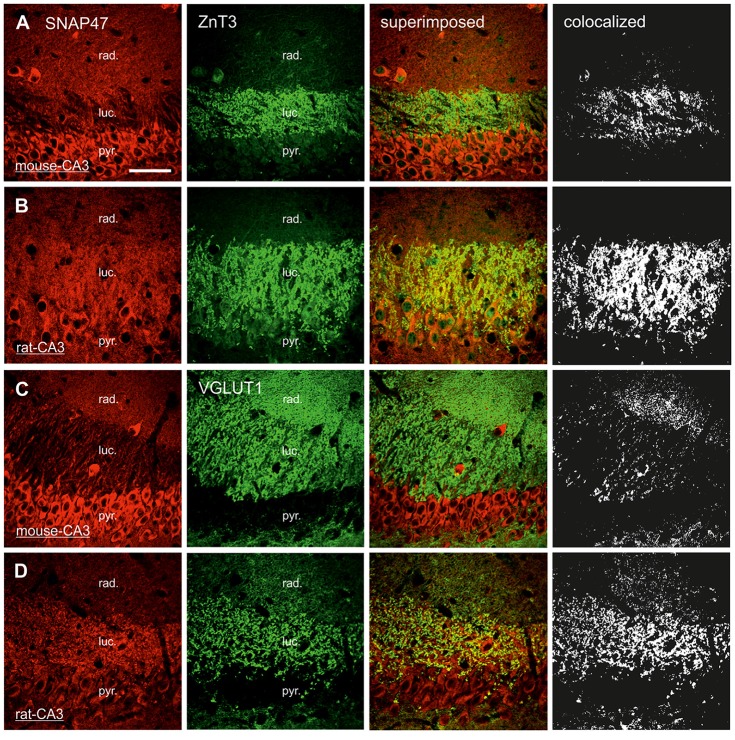
Presynaptic localization of SNAP47 in the CA3 area of mouse and rat hippocampus. **(A)** Confocal images of the co-localization of SNAP47 (red pseudocolor, left) and the presynaptic marker ZnT3 (in green, middle left) in the CA3 area of the mouse hippocampus. Note that SNAP47 shows weak labeling and weak co-localization with ZnT3 in the *stratum lucidum* corresponding to the mossy fiber (MF) terminals (see the superimposed signals and the colocalization on the rigth). **(B)** Confocal images of the co-localization of SNAP47 and the presynaptic marker ZnT3 in the CA3 area of rat hippocampus. The labeling for SNAP47 is strong in the *stratum lucidum* and shows substantial overlap with ZnT3. **(C)** Sections double stained against SNAP47 (red pseudocolor) and the presynaptic marker vesicular glutamate transporter 1 (VGLUT1; in green) in the CA3 area of the mouse hippocampus. **(D)** Sections double stained against SNAP47 and the presynaptic marker VGLUT1 in the CA3 area of the rat hippocampus. Scale bar represent, **(A–D)**, 50 μm.

**Figure 9:**
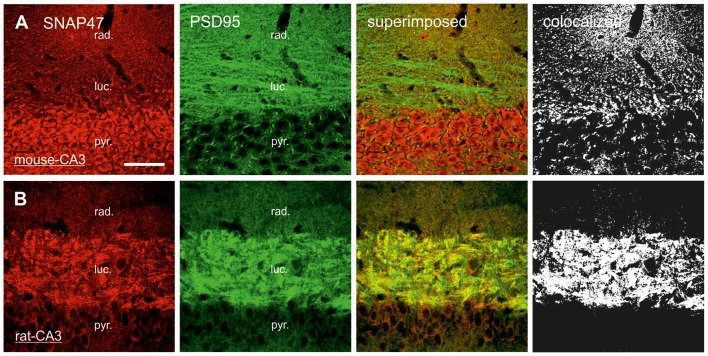
Postsynaptic localization of SNAP47 in the CA3 area of mouse and rat hippocampus. **(A)** Confocal images of the double immunolabeling for SNAP47 (red pseudocolor) and the postsynaptic marker PSD95 (in green) in the CA3 area of the mouse hippocampus. *Stratum lucidum* and *radiatum* show weak labeling for both SNAP47 and PSD95. Weak co-localization is observed in these two layers (see the superimposed signals and the colocalization on the rigth). **(B)** Double immunolabeling for SNAP47 and postsynaptic marker PSD95 in the CA3 area of the rat hippocampus. *Stratum lucidum* shows strong overlap of SNAP47 and PSD95. Scale bar represent, **(A,B)**, 50 μm.

In sections double stained for SNAP47 and ZnT3 (Figure 8B), ZnT3 showed a strong labeling in the CA3 *stratum lucidum* (Figure 8B) and the hilus was particularly strong at locations where MFBs were in close contact with somata and dendrites of CA3 pyramidal cells or putative hilar mossy cells. SNAP47 and ZnT3 labeling showed a partially overlapping pattern, indicating that SNAP47 was present presynaptically in MFBs in the rat hippocampus (Figure 8B). To confirm the results, we additionally performed double-immunolabeling for SNAP47 and VGLUT1, the most abundant presynaptic vesicular marker for glutamatergic synapses (Alonso-Nanclares et al., 2004). Indeed, we detected overlapping signals for VGLUT1 and SNAP47 in the MF termination zone in the CA3 *stratum lucidum* and the hilus of the rat (Figure 8D). In sections double-labeled for SNAP47 and PSD95, we observed a stronger overlap in the *stratum lucidum* and also in *stratum radiatum* of the CA3 (Figure 9B) as well as in the neuropil of the CA1 area, reflecting a preferential postsynaptic localization of the protein in both the MFB and the commissural/associational fiber systems of the rat.

#### Predominant Postsynaptic Labeling for SNAP47 in the Mossy Fiber Projection System in the Mouse

In contrast to the rat, the labeling for SNAP47 was low in the CA3 *stratum lucidum* of the mouse hippocampus. Accordingly, we found little co-localization of SNAP47 with ZnT3 (Figure 8A) or VGLUT1 presynaptically in MFBs (Figure 8C). Postsynaptically, however, SNAP47 showed a clear co-localization with PSD95 in the CA3 *stratum lucidum* of the mouse (Figure 9A). Furthermore, consistent with the stronger labeling intensity for SNAP47 in the CA3 *stratum radiatum* (Figure 8A), we found substantial colocalization of SNAP47 and PSD95 in this layer of the mouse hippocampus (Figure 9A). Similarly, colocalization of SNAP47 and PSD95 was strong in the CA1 *stratum radiatum*, too.

### Subcellular Localization of SNAP47

On the basis of our light microscopic analysis two major observations emerged: (1) SNAP47 is expressed at high levels in VGAT-positive inhibitory neurons in the adult mouse hippocampus; (2) in the rat hippocampus, the highest level of the immunolabeling was observed in excitatory MF system in putative postsynaptic elements. In order to confirm and further refine our confocal-microscopy observations, we have next performed postembedding immunogold labeling for SNAP47 combined with quantitative electron microscopic analysis.

#### Subcellular Localization of SNAP47 to Post- and Presynaptic Components of Glutamatergic MF Projection in the Rat Hippocampus

**Figure 10:**
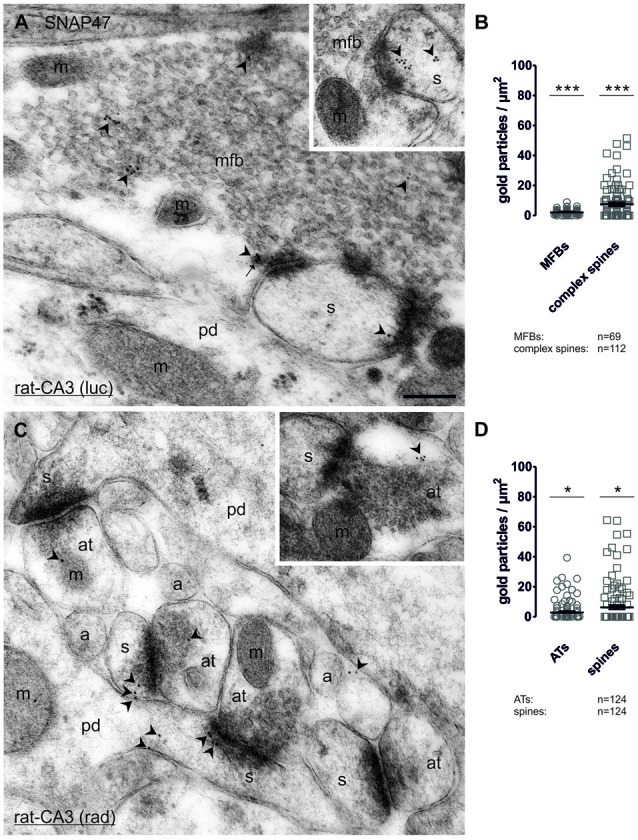
Quantitative immunoelectron microscopy of SNAP47 immunolabeling in glutamatergic neurons of the rat hippocampus. **(A)** Electron micrograph of a MF bouton (mfb) in the CA3 *stratum lucidum* labeled for SNAP47 (10 nm immunogold particles, arrowheads). The inset illustrates the high postsynaptic localization of SNAP47 in a spine head (s). Abbreviations: pd, pyramidal cell dendrite; m, mitochondrium; arrow, dense core vesicle. Scale bar: 500 nm. **(B)** Summary chart of the pre- and postsynaptic immunogold labeling density in the *stratum lucidum* of the CA3 area. Individual density values are superimposed for MFBs (open circles) and complex spines (open squares). **(C)** Postembedding immunogold labeling for SNAP47 in the CA3 *stratum radiatum*. Note that the immunogold labeling (arrowheads) is preferentially localized to spines (s) forming asymmetrical synapses with putative glutamatergic axon terminals (at), but was also observed in dendritic shafts of pyramidal cell dendrites (pd) and, at lower level, in axon terminal (inset). Abbreviations: a, axon. Scale bar: 500 nm. **(D)** Summary chart of pre- and postsynaptic immunogold labeling density in the *stratum radiatum* of the CA3 area. Individual density values are superimposed for axon terminals (AT, open circles) and spine heads (open squares). Asterisks indicate significant differences (***P < 0.0001 and *P < 0.02) relative to background labeling measured over mitochondria in the *stratum lucidum* **(B)** and *stratum radiatum* **(D)**.

In order to determine whether SNAP47 can, indeed, be localized to post- as well as presynaptic glutamatergic elements, as suggested by our immunofluorescence results, we next examined the MF projection in the rat hippocampus. MF boutons and their postsynaptic elements, the complex spines of CA3 pyramidal cells were identified on the basis of their ultrastructural features and localization in the *stratum lucidum*. MFBs show typical morphological characteristics, namely large presynaptic surface area, densely packed, clear, sphere-shaped SVs; a low number of dense core vesicles (DCVs); numerous mitochondria; numerous contacts with complex spines and *puncta adherentia* onto dendritic shafts (Frotscher et al., 1994). In ultrathin sections of CA3 *stratum lucidum*, immunogold-labeling for SNAP47 were present at high levels in complex spines engulfed by MFBs (Figure 10A and inset). However, the immunogold labeling was not confined to spines, but was also present in dendritic shafts, albeit at lower density. Furthermore, immunogold labeling was present presynaptically in MFBs in areas in close apposition to postsynaptic surface membranes of CA3 pyramidal cell dendrites, though at substantially lower density than on the postsynaptic side (Figure 10A). The labeling density was highly variable between MFBs (0–26 gold particles per MFB profile) and the distributions pattern of SNAP47 within MFBs seemed to be inhomogeneous, with clusters of immunogold particles associated to membrane structures within SV pools (Figure 10A).

Quantification of the labeling intensity confirmed these qualitative observations indicating that 19.7% of complex spines were clearly immunopositive (>2 particles per profile) with up to 15 gold particles per spine. In contrast, 33.9% of complex spines showed only 1–2 particles per profile and 46.4% were not labeled at all. The average density of immunogold particles was 7.4 ± 1.0 particle/μm^2^ over all complex spines (112 spines form two animals with an average area of 0.21 ± 0.02 μm^2^; Figure 10B). Presynaptically 76.9% were immunopositive (>2 particles per bouton), 13.4% of the MFBs showed labeling with 1–2 particles per bouton and 10.1% had no immunolabeling at all. Despite the high proportion of clearly immunopositive terminals, the mean labeling intensity with 2.1 ± 0.2 particle/μm^2^ (69 MFBs from two animals, mean cross-section area 3.28 ± 0.18 μm^2^, Figure 10B) was three-times lower than postsynaptically in complex spines. The density of immunogold particles measured in both pre- and postsynaptic compartments was significantly higher compared to the background (*P* < 0.0001 for both compartments; Figure 10B) measured over mitochondria in MFBs.

To further test whether the strong, but sparse postsynaptic localization of SNAP47 applied to other glutamatergic synapses, we also took samples of small glutamatergic boutons in *stratum radiatum* of the CA3 area. These asymmetrical synapses were identify by the close apposition of a small presynaptic boutons filled with small clear SVs and a postsynaptic dendritic spine with strong postsynaptic density (PSD) separated by a well-defined synaptic cleft (Figure 10C and inset). At these synapses gold labeling was much lower than in MFBs and was present only in a total of 6.5% (≥2 particles per profile) postsynaptically and 9.7% (≥2 particles per bouton) presynaptically. The mean density of immunogold particles postsynaptically, in spines was 6.2 ± 1.3 particle/μm^2^ (124 spines form two animals with an average area of 0.06 ± 0.004 μm^2^; Figure 10D) whereas it was two-times lower at 2.8 ± 0.6 particle/μm^2^ presynaptically, in small putative glutamatergic axon terminals (124 terminals from two animals with an average area of 0.14 ± 0.01 μm^2^; Figure 10D). The density of immunogold particles postsynaptically (*P* = 0.0101) and presynaptically (*P* = 0.0151; Figure 10D) was significantly higher than the background obtained over mitochondria in the *stratum radiatum*.

In summary, our quantification documented an enrichment of SNAP47 postsynaptically in spines and additionally revealed quantitative differences in the expression across the layers of the CA3 area, with higher labeling present in complex spines in the *stratum lucidum* as opposed to small dendritic spines in the *stratum radiatum*. Moreover, labeling for SNAP47 was clearly present in most presynaptic terminals examined, but at a level lower than that detected in postsynaptic compartments.

#### Subcellular Localization of SNAP47 to Post- and Presynaptic Components of GABAergic INs in the Mouse

**Figure 11:**
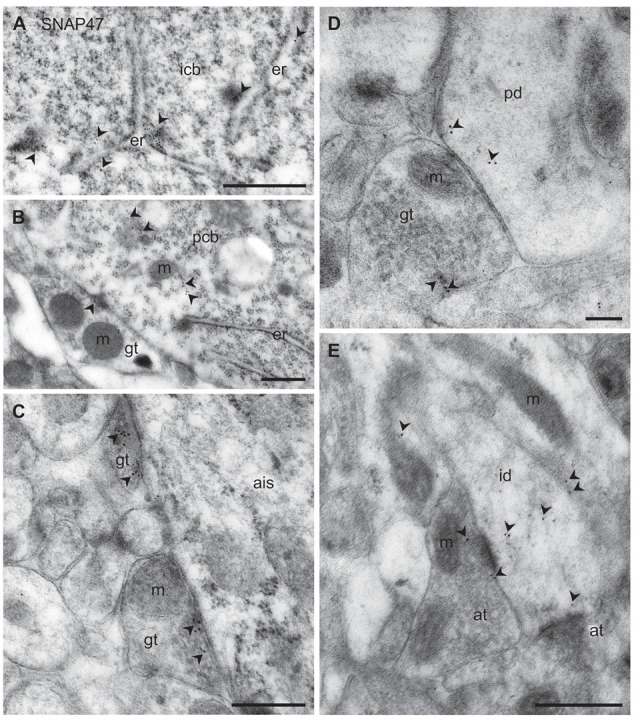
Ultrastructural localization of SNAP47 in axon terminals and dendrites of GABAergic INs of the mouse hippocampus. **(A)** Postembedding immunogold labeling for SNAP47 (10 nm gold, arrowheads) in the cytoplasm of a putative interneuron cell body (icb) in the CA3 *stratum radiatum* associated to the endoplasmic reticulum (ER). Scale bar: 500 nm. **(B)** A putative GABAergic basket cell terminal (GABAergic terminals, gt) forming symmetric contact onto a pyramidal cell body (pcb) in the CA3 *stratum pyramidale*. Note that postembedding immunogold labeling for SNAP47 (10 nm gold; arrowheads) was preferentially found postsynaptically in the pyramidal cell body and only at lower level presynaptically in the inhibitory axon terminals. Scale bar: 200 nm. **(C)** Putative gt making symmetric contacts with the axon initial segment (AIS) of a pyramidal cell in the CA3 region. Arrowheads indicate immunogold labeling for SNAP47 (10 nm gold) found at low level in the axon terminals. Scale bar: 500 nm. **(D)** Putative gt making symmetric contact with a dendritic shaft (pd) of a putative pyramidal cell in the CA3 region. Arrowheads indicate immunogold labeling (10 nm gold) for SNAP47 found in pyramidal dendritic shaft and at low level in the axon terminal. Scale bar: 200 nm. **(E)** A putative interneuron dendritic shaft (id) receiving convergent asymmetrical, presumably excitatory synapses with small axon terminals (at) in the CA1 *stratum radiatum*. Arrowheads indicate immunogold labeling (10 nm gold) for SNAP47 found in the dendritic shaft and in the axon terminal. Scale bar: 500 nm.

Consistent with the strong immunofluorescence labeling for SNAP47 in VGAT-Venus INs in the mouse hippocampus, immuno-electron microscopy showed high levels of immunogold particles for the protein at the membrane of the endoplasmic reticulum (ER) in cell bodies of VGAT-positive INs (Figure 11A). Similar to INs, somata of putative PCs in the CA1 *stratum pyramidale* showed high levels of immunogold particles associated to the ER (Figure 11B). This localization plausibly reflects a reserve pool, related to production and trafficking of the protein in these neurons.

To address the subcellular distribution of functional protein at pre- and postsynaptic membranes, we next examined immunolabeling for the protein presynaptically in well-defined GABAergic synapse populations: (1) putative basket cell terminals forming symmetric synaptic contacts with cell bodies of principal cells (Figure 11B); (2) axo-axonic synapses on the axon initial segment (AIS) of principal cells (Figure 11C); and (3) axo-dendritic inhibitory synapses forming contact onto dendritic shafts of principal cells in the CA1 and CA3 area (Figure 11D). All types of inhibitory synapses were identified based on their symmetric contact to the postsynaptic component. In all these three major inhibitory presynaptic axon terminals, we could occasionally detect labeling for SNAP47, suggesting that SNAP47 could be trafficked to presynaptic sites also in inhibitory neuron populations. Finally, we investigated labeling postsynaptically, in IN dendrites in the neuropil. Indeed, immunogold particles for SNAP47 protein were consistently found to be localized, albeit at low levels, to non-spiny dendritic shafts of putative INs receiving convergent asymmetrical, presumably excitatory synapses in the *stratum radiatum* of the CA1 area (Figure 11E).

Thus, the immunogold labeling pattern of SNAP47 in INs observed in the electron microscope indicates trafficking of this protein to both pre- and postsynaptic compartments.

## Discussion

The present study reveals a divergent distribution of SNAP47 in mouse and rat hippocampus. While immunolabeling for SNAP47 is broadly distributed in all areas and layers of the hippocampus in both the mouse and rat, it shows an almost complementary staining pattern. In the mouse the strongest labeling was observed in VGAT-positive inhibitory INs of the hippocampus, whereas in the rat hippocampus the highest level of immunostaining was observed in the termination zone of the excitatory MF projection. Independent of these differences in areal and cellular distribution, at the subcellular level the protein was found to be localized to both postsynaptic and presynaptic elements, with higher densities in postsynaptic elements.

### High Expression of the SNAP47 in Somata of GABAergic INs in the Mouse

Immunofluorescence labeling for SNAP47 revealed a strong expression of this protein in cell body layers of the mouse hippocampus. Furthermore, particularly high labeling was observed in somata scattered in all areas and layers corresponding to inhibitory GABAergic INs. This finding is in good agreement with previous documented localization of this protein in cell bodies of cultured striatal neurons (Holt et al., 2006). Although the precise subcellular localization could not be identified in that study, our postembedding immunogold data suggest that the protein is closely associated to the ER in both GABAergic VGAT-positive and glutamatergic pyramidal neuron populations. This pool could recruit factors involved in the regulation of the structural organization of the ER or Golgi-ER and post-Golgi transport possibly through interaction with cytoskeletal elements (Kuster et al., 2015), but the precise role remains to be established.

The high accumulation of SNAP47 in inhibitory GABAergic neurons of the adult mouse hippocampus was an unexpected finding. Previously, Tafoya et al. (2006) reported a high expression of SNAP25 in GABAergic terminals (gt) of the hippocampus and the thalamus, however this finding was contrasted by several reports suggesting the lack of SNAP25 in hippocampal GABAergic axon terminals (Verderio et al., 2004; Bragina et al., 2007; Matteoli et al., 2009). In contrast, SNAP23 has been reported to be highly and selectively expressed in at least a subset of hippocampal and neocortical inhibitory axon terminals (Verderio et al., 2004; Bragina et al., 2007). Our results indicate that GABAergic neurons in the mouse hippocampus synthesize SNAP47 at high levels and the functional protein can be localized both postsynaptically in somato-dendritic compartments and presynaptically in axon terminals. In the rat somatic expression levels were markedly lower than in the mouse, albeit not absent, suggesting that the storage and trafficking of this SNAP isoform underlies species-dependent regulatory mechanisms. Further investigations are necessary to identify the precise localization and role of the functional protein in diverse IN populations and whether, despite a low presynaptic expression, it can participate in the SNARE complexes involved in supporting vesicle fusion in GABAergic boutons.

### Post- and Presynaptic Expression of SNAP47 at Glutamatergic Synapses

In the rat hippocampus, SNAP47 immunolabeling of the cell body layers was weak, whereas the neuropil, in particular the CA3 *stratum lucidum* where MFs terminate showed strong staining. We could detect SNAP47 at high level in subset of dendritic spines, and showed localization similar to the postsynaptic marker. This finding is consistent with the conclusion from a previous study demonstrating that SNAP47 is present in postsynaptic compartments and an essential component of the fusion machinery required for regulated AMPAR insertion to the membrane during the expression of LTP (Jurado et al., 2013). However, the protein does not contribute to the regulation of basal AMPAR- or NMDAR-mediated synaptic responses or basal AMPAR surface expression (Jurado et al., 2013). The study by Jurado et al. (2013) did not allow any conclusions about the specific dendritic nanodomains at which AMPAR exocytosis occurs during LTP or about the specific timing of these events. Other studies have found that following NMDAR activation, recombinant AMPAR subunits are inserted into perisynaptic membranes, either adjacent to the PSD or adjacent to the base of dendritic spines, from where they can laterally diffuse into the PSD to increase synaptic strength (Petrini et al., 2009; Opazo et al., 2010). The sources of the AMPARs that are exocytosed during LTP have been suggested to be recycling endosomes containing transferrin receptors (TfRs) in dendrite spines (Jurado et al., 2013). As there is a close correlation between dendritic spine size and PSD size (Harris and Stevens, 1989) and between PSD size and AMPAR density at synapses (Takumi et al., 1999), small spines in the *stratum radiatum* are likely to contain lower numbers of AMPARs. Assuming that SNAP47 expression is proportional to AMPAR numbers, this could explain the higher intensity of SNAP47 labeling in complex spines in CA3 *stratum lucidum* in comparison to spines in CA3 *stratum radiatum* in rat hippocampus, as observed in our study. In general, predominant localization of SNAP47 to the postsynaptic elements supports role of this protein in the control of receptor trafficking.

In our study, we could also confirm presynaptic localization of SNAP47 in glutamatergic synapses. The labeling intensity was particularly high in MFBs in CA3 *stratum lucidum* in the rat. We could detect co-localization of SNAP47 immunofluorescence signal with both VGLUT1- and ZnT3 in MFBs, but not all MFBs were labeled and the overlap was weaker than with the postsynaptic marker. In fact, postembedding immunogold-labeling confirmed these findings and demonstrated that SNAP47 immunogold particles were present presynaptically in the majority of MFBs however at substantially lower densities than postsynaptically in the complex spines of CA3 pyramidal cells.

The presynaptic localization of SNAP47 in MFBs may have important implications in vesicular exocytosis of BDNF (Shimojo et al., 2015). BDNF is stored in discrete vesicles within MFBs and some MFB-filopodia (Danzer et al., 2004; Dieni et al., 2012). BDNF-containing clusters co-localize neither with SV markers such as synaptophysin or VGLUT1, nor with proteins that are stored in dense core vesicle fractions. In good agreement, the staining pattern for SNAP47 was different from established markers for synapses. Furthermore, the co-fractionation pattern indicates that a pool of SNAP47 resides on a subpopulation of small vesicles, but the nature and origin of these vesicles remains to be established (Holt et al., 2006). Synaptic BDNF is not uniquely stored in MFBs, but was generally found in discrete synaptic granules/vesicles in majority of glutamatergic axon terminals in cultured hippocampal neurons (Andreska et al., 2014). Based on our results which show irregular, clustered localization of SNAP47 within glutamatergic presynaptic boutons, different from the homogenous vesicular labeling for VGLUT1, we hypothesize a co-localization of BDNF and SNAP47. Moreover, as BDNF protein is further localized in cell bodies (Conner et al., 1997) and SNAP47 protein is also accumulated in neuronal (glutamatergic and GABAergic) cell bodies. This co-localization pattern seems not to be restricted to MFBs, but may be a general principal for all neuronal compartments.

In summary, our work shows that SNAP47 is present postsynaptically at high levels in a subset of postsynaptic elements, including CA3 complex spines in the *stratum lucidum* and small spines in the CA1 and CA3 *stratum radiatum*. However, SNAP47 is also present presynaptically at lower levels in subsets of axon terminals, including MFBs and small excitatory axon terminals in the *stratum radiatum*. Concluding, our data suggest that SNAP47 may be involved not only in postsynaptic function but also in unique fusion machinery, distinct from the one involved in neurotransmitter release at the presynaptic sites.

### Future Perspectives

With respect to the strong accumulation of SNAP47 in GABAergic cell bodies and the co-localization of GABA pre- and postsynaptic markers with this SNAP isoform examined in the present study, it will be of interest to clarify its precise cellular and subcellular expression in various types of inhibitory neurons and to identify the mechanisms that regulate its intracellular distribution and trafficking and will helps us better understand the functions of SNAP47 protein in GABAergic neurons. The preferential localization of SNAP47 to the postsynaptic components in the rat at physiological conditions raises the question whether the trafficking of this protein, in terms of favored localization to the presynaptic terminals and/or postsynaptic sites, could be changed dynamically during development or brain disorders.

## Author Contributions

AM-W and IV designed the experiments and analyzed the data; HH performed immunohistochemical labeling experiments; FB performed confocal image acquisition and quantification of immunofluorescence; TT prepared lentivirus construct, western blot analysis and all probes for *in situ* hybridization; HH and AM-W performed *in situ* hybridization; YY generated VGAT-Venus transgenic mice and rats; AM-W performed electron microscopic imaging and quantification; AM-W and IV prepared the manuscript.

## Conflict of Interest Statement

The authors declare that the research was conducted in the absence of any commercial or financial relationships that could be construed as a potential conflict of interest.

## Footnotes

^1^http://rsb.info.nih.gov/ij/
