# Supplementary material for: Distinct Localization of SNAP47 Protein in GABAergic and Glutamatergic Neurons in the Mouse and the Rat Hippocampus
Source: Front Neuroanat. 2017 Jul 13;11:56. doi: 10.3389/fnana.2017.00056 (PMC5508021; doi:10.3389/fnana.2017.00056)
Supplement: Supplementary file 1 [file Presentation_1.pdf]

Supplementary Information for „Distinct localization of SNAP47 protein in GABAergic and glutamatergic neurons in the mouse and the rat hippocampus“

Agnieszka Münster-Wandowski[1]*, Heike Heilmann[1], Felix Bolduan[1], Thorsten Trimbuch[3], Yuchio Yanagawa[4], Imre Vida[1,2]*

[1]Institute of Integrative Neuroanatomy, Charité - Universitätsmedizin Berlin, Campus Mitte, Berlin, Germany
[2]Neurocure Cluster of Excellence, Charité - Universitätsmedizin Berlin, Campus Mitte, Berlin, Germany
[3]Institute of Neurophysiology, Charité - Universitätsmedizin Berlin, Campus Mitte, Berlin
[4]Departments of Genetic and Behavioral Neuroscience, Gunma University, Graduate School of Medicine, Maebashi City, Japan

*Correspondence:
Dr. Agnieszka Münster-Wandowski
Institute of Integrative Neuroanatomy
Charité - Universitätsmedizin Berlin
Virchowweg 6, CCM, 10117 Berlin
Tel. +49 30 450 528 264
agnieszka.muenster-wandowski@charite.de

Prof. Dr. Imre Vida
Institute of Integrative Neuroanatomy
Charité - Universitätsmedizin Berlin
Virchowweg 6, CCM, 10117 Berlin
Tel. +49 30 450 528 118
imre.vida@charite.de

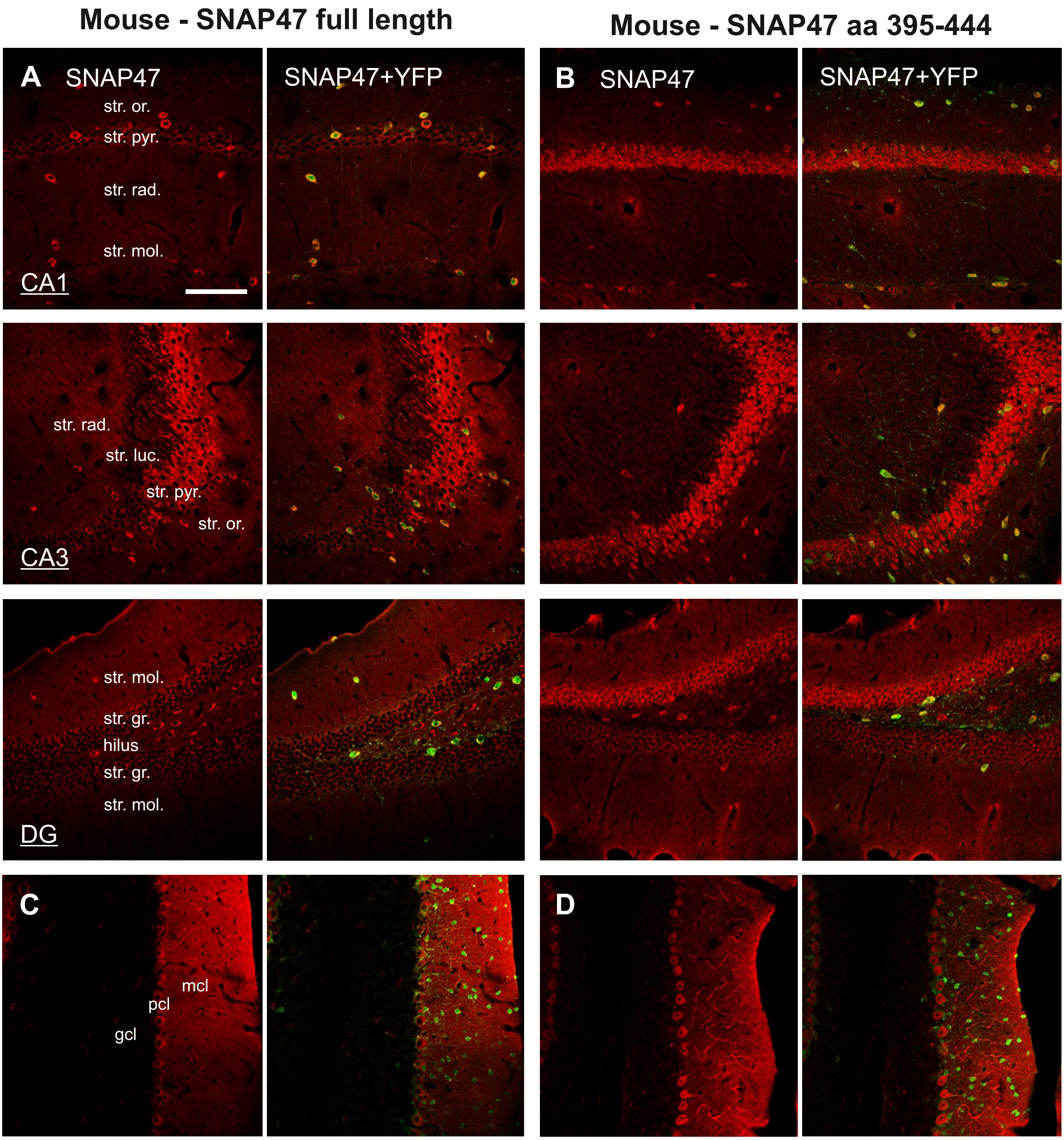

Supplementary Figure 1. Convergent immunofluorescent labeling pattern obtained with two different SNAP47 antibodies in the hippocampus and cerebellum of the Venus(YFP) mouse

A: Overview confocal images of double immunolabeling for SNAP47 (in red, left images) and YFP (in green, merged images on the right) in the hippocampal CA1 (top row), the CA3 (middle row) and DG (bottom row) obtained with the polyclonal antibody raised against recombinant full length SNAP47 available from SySy (used in our study) in the mouse.

B: Overview images of double immunolabeling for SNAP47 (in red, left images) and YFP (in green, merged images on the right) obtained with antibody raised against SNAP47 synthetic peptide aa395- 444 available from Abcam. Note the high similarity of the immunolabeling pattern in all 3 hippocampal areas and that the scattered YFP positive interneurons are positive for SNAP47.

C, D: Overview confocal images of double immunolabeling for SNAP47 (in red, left images) and YFP (in green, merged images on the right) in the cerebellum obtained with the polyclonal antibody raised against recombinant full length SNAP47 (C) and the synthetic peptide aa395-444 (D).

Scale bar represent, A-D, 100 µm

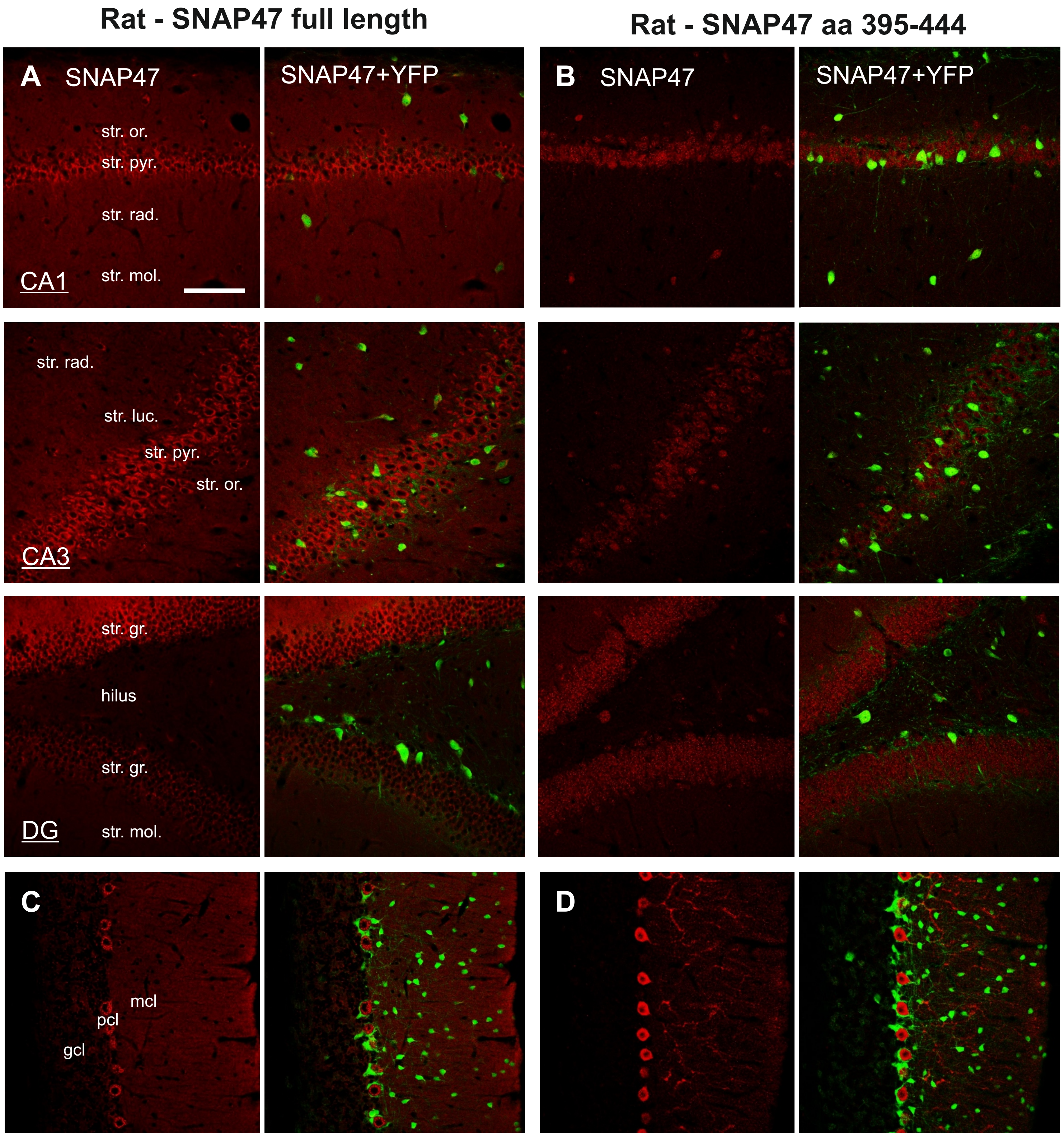

Supplementary Figure 2. Convergent immunofluorescent labeling pattern obtained with two different SNAP47 antibodies in the hippocampus and cerebellum of the Venus(YFP) rat

A: Overview confocal images of double immunolabeling for SNAP47 (in red, left images) and YFP (green, merged images on the right) in the hippocampal CA1 (top row), the CA3 (middle row) and DG areas (bottom row) obtained with the polyclonal antibody raised against recombinant full length SNAP47 available from SySy (used in our study) in the rat.
B: Overview images of double immunolabeling for SNAP47 (in red, left images) and YFP (in green, merged images on the right) obtained with antibody raised against SNAP47 synthetic peptide aa395- 444 available from Abcam. Note the high similarity of the immunolabeling pattern in all 3 hippocampal areas and that the some scattered YFP positive interneurons are positive for SNAP47.
C, D: Overview confocal images of double immunolabeling for SNAP47 (in red, left images) and YFP (in green, merged images on the right) in the cerebellum obtained with the polyclonal antibody raised against recombinant full length SNAP47 (C) and the synthetic peptide aa395-444 (D).
Scale bar represent, A-D, 100 μm

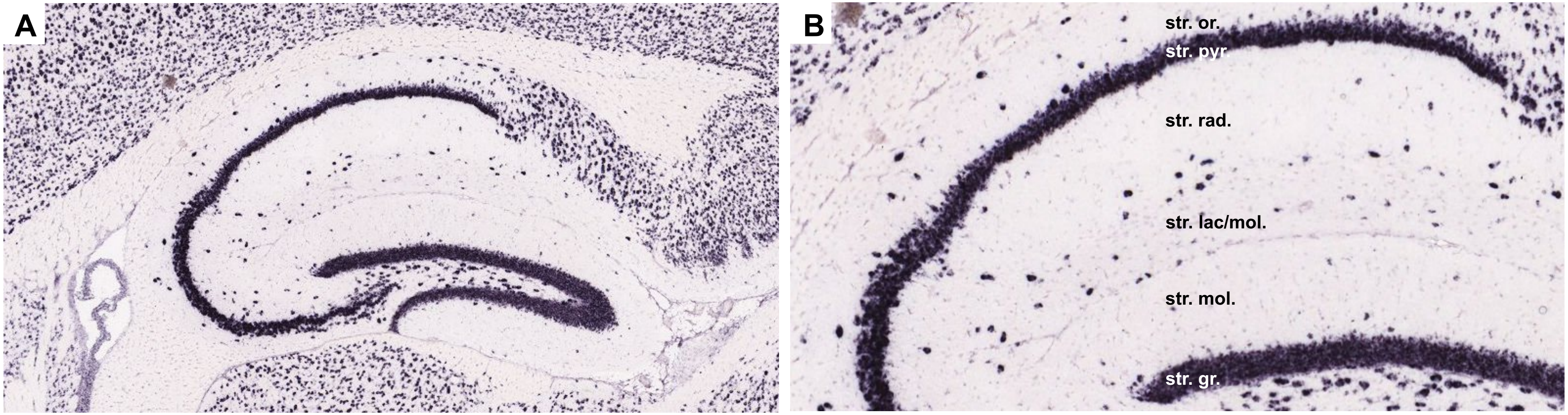

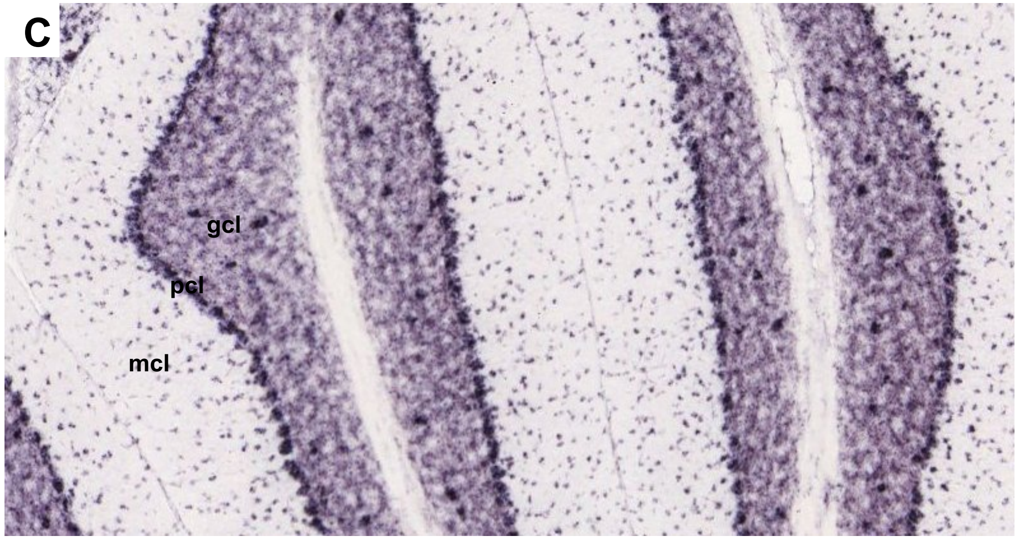

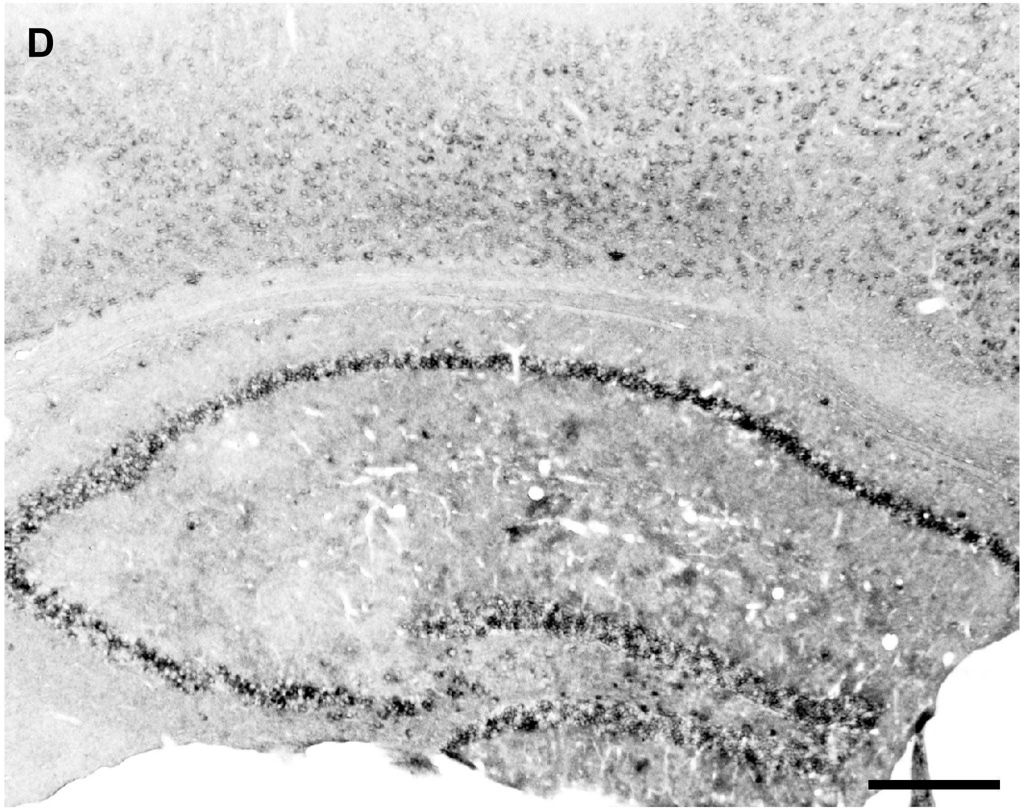

Supplementary Figure 3: RNA-ISH labeling for SNAP47 in the mouse hippocampus and cerebellum

A, B: in situ hybridization images of SNAP47 RNA expression in hippocampus. Note the strong expression of SNAP47 RNA in hippocampal cell layers and scattered neurons over all layers.
C: in situ hybridization images of SNAP47 RNA expression in cerebellum. Note the strong expression of SNAP47 RNA in hippocampal cell layers and scattered population of putative interneurons (A, B) and the intense labeling of Purkinje neurons in cerebellum (C). (A, B, C: Images from the Allen Mouse Brain Atlas; courtesy of the Allen Institute, available from: http://mouse.brain-map.org/experiment/show?id=76098395; Lein et al., 2007).
D: Overview image in situ hybridization with anti-sense probe in Venus(YFP) mouse hippocampus. Note the strong expression of SNAP47 RNA in pyramidal and granular cell layers and scattered putative interneurons in all layers. Scale bar represent 100 µm in D.
